# The economic impact of pandemic influenza in the United States: priorities for intervention.

**DOI:** 10.3201/eid0505.990507

**Published:** 1999

**Authors:** M I Meltzer, N J Cox, K Fukuda

**Affiliations:** National Center for Infectious Diseases, Centers for Disease Control and Prevention, Atlanta, GA 30333, USA. qzm4@cdc.gov

## Abstract

We estimated the possible effects of the next influenza pandemic in the United States and analyzed the economic impact of vaccine-based interventions. Using death rates, hospitalization data, and outpatient visits, we estimated 89,000 to 207,000 deaths; 314,000 to 734,000 hospitalizations; 18 to 42 million outpatient visits; and 20 to 47 million additional illnesses. Patients at high risk (15% of the population) would account for approximately 84% of all deaths. The estimated economic impact would be US$71.3 to $166.5 billion, excluding disruptions to commerce and society. At $21 per vaccinee, we project a net savings to society if persons in all age groups are vaccinated. At $62 per vaccinee and at gross attack rates of 25%, we project net losses if persons not at high risk for complications are vaccinated. Vaccinating 60% of the population would generate the highest economic returns but may not be possible within the time required for vaccine effectiveness, especially if two doses of vaccine are required.


### The Economic Impact of Pandemic Influenza in the United States: Priorities for Intervention

**Martin I. Meltzer, Nancy J. Cox, and Keiji Fukuda**
Centers for Disease Control and Prevention, Atlanta, Georgia, USA

We estimated the possible effects of the next influenza pandemic in the United States and analyzed the economic impact of vaccine-based interventions. Using death rates, hospitalization data, and outpatient visits, we estimated 89,000 to 207,000 deaths; 314,000 to 734,000 hospitalizations; 18 to 42 million outpatient visits; and 20 to 47 million additional illnesses. Patients at high risk (15% of the population) would account for approximately 84% of all deaths. The estimated economic impact would be US$71.3 to $166.5 billion, excluding disruptions to commerce and society. At $21 per vaccinee, we project a net savings to society if persons in all age groups are vaccinated. At $62 per vaccinee and at gross attack rates of 25%, we project net losses if persons not at high risk for complications are vaccinated. Vaccinating 60% of the population would generate the highest economic returns but may not be possible within the time required for vaccine effectiveness, especially if two doses of vaccine are required.

Influenza pandemics have occurred for centuries, three times (1918, 1957, and 1968) in the 20th century alone. Another pandemic is highly likely, if not inevitable (1). In the 1918 influenza pandemic, more than 20 million people died (2). Improvements in medical care and technology since the last pandemic may reduce the impact of the next. When planning for the next pandemic, however, decision makers need to examine the following questions: Would it make economic sense to vaccinate the entire U.S. population if 15% were to become clinically ill? What if 25% were to become ill? To answer such questions, we conducted economic analyses of potential intervention scenarios.

Although many studies have examined or reviewed the economics of influenza vaccination (3-10), only one study (11), published in 1976, examined the economics of a vaccine-based intervention aimed at reducing the impact of an influenza epidemic in the United States. Our study examines the possible economic effects of the next influenza pandemic in the United States, analyzes these effects, and uses the results to estimate the costs, benefits, and policy implications of several possible vaccine-based interventions. These estimates can be used in developing national and state plans to respond to an influenza pandemic.[1] Unlike the 1976 study, ours examined the effect of varying the values of a number of key input variables. Specific objectives were to provide a range of estimates regarding the number of deaths, hospitalizations, outpatient visits, and those ill persons not seeking medical care in the next influenza pandemic; provide a cost estimate of health outcomes; estimate the potential net value of possible vaccination strategies;[2] evaluate the effect of using different criteria (e.g., death rates, economic returns due to vaccination) to set vaccination priorities; assess the economic impact of administering various doses of vaccine

Address for correspondence: Martin Meltzer, National Center for Infectious Diseases, Centers for Disease Control and Prevention, Clifton Road, Mail Stop C12, Atlanta, GA 30333, USA; fax: 404-639-3039; e-mail: qzm4@cdc.gov.

[1]A complete plan detailing a response to an influenza pandemic should include definition of a pandemic, points that will initiate various steps in the response plan, and details about deploying the intervention. While a U.S. federal influenza pandemic plan is being developed, a guide to aid state and territorial health officials in developing plans for their jurisdictions is available at http://www.cdc.gov/od/nvpo/pandemicflu.htm. Printed copies can be obtained from the author.

[2]We limited our examination of possible interventions to those involving influenza vaccines. We did not consider the use of antiviral drugs for influenza prophylaxis because there may not be adequate supplies; first priority for such drugs may be for treatment; and the side-effects from the drugs, particularly amantadine, make them unsuitable for long-term prophylaxis for many workers, such as drivers, or heavy construction operators.

and of administering vaccine to different age groups and groups at risk; and calculate an insurance premium that could reasonably be spent each year for planning, preparedness, and practice.

### Methods

### The Model

Building a mathematical model of the spread of influenza is difficult largely because of differences in virus transmission and virulence, lack of understanding of the primary factors affecting the spread of influenza, and shortage of population-based data (12). Because of the difficulties in calculating realistic estimates of the numbers of cases in the next influenza pandemic, we used a Monte Carlo mathematical simulation model (13-15), which uses predefined probability distributions of key input variables to calculate the number of illnesses and deaths that could result from an influenza pandemic. Some of the most important probability distributions we used describe the population-based rates of illness and death. These rates are based on illness and death rates reported in earlier influenza pandemics and epidemics. The model produces a range of estimated effects rather than a single point estimate. The model is not epidemiologic and thus does not describe the spread of the disease through a population.

Many details of the model are presented below and in Appendix I; a more detailed explanation and a complete list of all the variables used and the values assigned to the variables are available at Appendix II.

For interventions to contain and reduce the impact of an influenza pandemic, we used a societal perspective, which takes into account all benefits and all costs regardless of who receives and who pays.

### Age Distribution and Persons at High Risk

Since the age distribution of patients in the next pandemic is unknown, we assumed a distribution (Table 1) among the three age groups (0 to 19 years, 20 to 64 years, and 65 years and older).[3] Further, each age group was divided into those at high risk (persons with a preexisting medical condition making them more susceptible to complications from influenza) and those not at high risk (Table 1).[4] Age by itself was not considered a risk factor; persons 65 years and older were assumed to have higher rates of illness and death than the rest of the population (Table 2).

Table 1. Estimate of age distribution of cases and percentage of population at high risk used to examine the impact of pandemic influenza in the United States

| Age group (yrs) | Percentage of all cases[a] |
|---|---|
| 0-19 | 40.0 |
| 20-64 | 53.1 |
| 65 + | 6.8 |
| Totals[b] | 100.0 |
| | Percentage at high risk[c] |
| 0-19 | 6.4 |
| 20-64 | 14.4 |
| 65 + | 40.0 |
| U.S. average[d] | 15.4 |

[a]The actual number of cases will depend upon the assumed gross attack rate. The distribution of cases was based on lower and upper estimates of age-specific attack rates from the 1918, 1928-29, and 1957 epidemics and pandemics (19).
[b]Totals do not add to exactly 100% because of rounding.
[c]Persons are categorized at high risk if they have a preexisting medical condition that makes them more susceptible to influenza-related complications. The percentages of age groups at high risk were obtained from the Working Group on Influenza Pandemic Preparedness and Emergency Response (GrIPPE, unpub. data). The Advisory Committee on Immunization Practices estimates that 27 to 31 million persons aged <65 years are at high risk for influenza-associated complications (17).
[d]Average is an age-weighted average, using each age group's proportion of the total U.S. population.

[3]This article presents the results for one distribution of cases by age and risk group. The background paper in Appendix II, however, contains additional results obtained by using a different distribution.
[4]The Advisory Committee on Immunization Practices estimates that 27 to 31 million people ages <65 years are at high risk for influenza-associated complications (17). ACIP also classifies all 32 million people ≥65 years as being at elevated risk for influenza-related complications (17). Further, the working group on influenza pandemic preparedness and emergency response has assumed that approximately 19 million household members of persons at high risk should also be vaccinated to reduce the probability of transmission to those at high risk (GrIPPE, unpub. data, 1997).

### Gross Attack Rates

In the model, we used gross attack rates (percentage of clinical influenza illness cases per population) of 15% to 35%, in steps of 5%. Infected persons who continued to work were not considered to have a clinical case of influenza, and were not included.

### Illnesses and Deaths

The rates of adverse effects (outpatient visits, hospitalizations, deaths, and illnesses for which no medical care was sought), by age and risk group, were used to determine the number of persons in each category (Table 2) (Appendix II).

Table 2. Variables used to define distributions of disease outcomes of those with clinical cases[a] of influenza

| | Rates per 1,000 persons[b] | | |
|---|---|---|---|
| Variable | Lower | Most likely | Upper |
| Outpatient visits | | | |
| Not at high risk | | | |
| 0-19 yrs old | 165 | | 230 |
| 20-64 yrs old | 40 | | 85 |
| 65 + yrs old | 45 | | 74 |
| High risk | | | |
| 0-19 yrs old | 289 | | 403 |
| 20-64 yrs old | 70 | | 149 |
| 65 + yrs old | 79 | | 130 |
| Hospitalizations | | | |
| Not at high risk | | | |
| 0-19 yrs old | 0.2 | 0.5 | 2.9 |
| 20-64 yrs old | 0.18 | | 2.75 |
| 65 + yrs old | 1.5 | | 3.0 |
| High risk | | | |
| 0-19 yrs old | 2.1 | 2.9 | 9.0 |
| 20-64 yrs old | 0.83 | | 5.14 |
| 65 + yrs old | 4.0 | | 13 |
| Deaths | | | |
| Not at high risk | | | |
| 0-19 yrs old | 0.014 | 0.024 | 0.125 |
| 20-64 yrs old | 0.025 | 0.037 | 0.09 |
| 65 + yrs old | 0.28 | 0.42 | 0.54 |
| High risk | | | |
| 0-19 yrs old | 0.126 | 0.22 | 7.65 |
| 20-64 yrs old | 0.1 | | 5.72 |
| 65 + yrs old | 2.76 | | 5.63 |

[a]Clinical cases are defined as cases in persons with illness sufficient to cause an economic impact. The number of persons who will be ill but will not seek medical care, are calculated as follows: Number $\text{ill}_{age}$ = ($\text{Population}_{age}$ x gross attack rate) - ($\text{deaths}_{age}$ + $\text{hospitalizations}_{age}$ + $\text{outpatients}_{age}$). The number of deaths, hospitalizations, and outpatients are calculated by using the rates presented in this table.

[b]For Monte Carlo simulations, rates are presented as lower and upper for uniform distributions, and lower, most likely, and upper for triangular distributions (18).

Sources: 3,6,11,19-29, and Appendix II.

### Net Returns of Vaccinating against an Influenza Pandemic

Vaccinating predefined segments of the population will be one of the major strategies for reducing the impact of pandemic influenza, and the net return, in dollars, from vaccination is an important economic measure of the costs and benefits associated with vaccination. We calculated the net return by using the following formula for each age and risk group:

$$\text{Net returns}_{\text{age, risk group}} = \text{Savings from outcomes averted in population}_{\text{age, risk group}} - \text{cost of vaccination of population}_{\text{age, risk group}}$$

The savings from illnesses and deaths averted and the cost of vaccinations are described in Appendix I. Some input variables are described below and in Appendix II.

### Input Variables

The direct medical costs (i.e., those reimbursed by third-party payers such as health insurance companies) associated with hospitalizations, outpatient visits, and drug purchases were obtained from a proprietary database containing health insurance claims data from approximately 4 million insured persons (The MEDSTAT Group, Ann Arbor, MI) (Table 3). Following the methods used by McBean et al. (28), we extracted the data for outpatient visits from the database with codes from the International Classification of Diseases, Ninth Revision (ICD-9) for pneumonia and bronchitis (ICD-9: 480-487.8), acute bronchitis (ICD-9: 466-466.1), and chronic respiratory disease (ICD-9: 490-496). Costs for inpatient care were extracted with the same codes, when recorded as the principal diagnosis and when recorded as any of the diagnoses in a patient's chart. Further, because influenza can cause patients with preexisting medical conditions to seek inpatient care, data were extracted for the inpatient costs of treating heart-related conditions (common preexisting conditions that place a person at high

Table 3. Input variables used to calculate the economic impact (direct and indirect costs) of health outcomes due to an influenza pandemic in the United States (in 1995 US$)

| Outcome category item | Type of cost | Age group (yrs) 0-19 | 20-64 | 65+ | Sources |
|---|---|---|---|---|---|
| **Deaths** | | | | | |
| Average age (years) | | 9 | 35 | 74 | Assumed |
| PV earnings lost ($)[a] | Indirect | 1,016,101 | 1,037,673 | 65,837 | 16, 30 |
| Most likely ± min or max hospital costs ($)[b] | Direct | 3,435±2,632 | 7,605±3,888 | 8,309+3,692 | Marketscan Database; 31. |
| Subtotal ($)[c] | | 1,019,536 | 1,045,278 | 74,146 | |
| **Hospitalizations** | | | | | |
| Most likely ± min or max hospital costs ($)[b] | Direct | 2,936±2,099 | 6,016±2,086 | 6,856±3,200 | Marketscan Database; 31. |
| Most likely ± min or max net pay for outpatient visits ($)[d] | Direct | 74 ± 40 | 94 ± 70 | 102 ± 60 | Marketscan Database; 31. |
| Avg. copayment for outpatients visit ($) | Direct | 5 | 4 | 4 | Marketscan Database |
| Most likely ± min or max net payment for drug claims($)[e] | Direct | 26 ± 9 | 42 ± 30 | 41 ± 10 | Marketscan Database |
| Most likely ± min or max days lost[f] | Indirect | 5 ± 2.7 | 8 ± 4.8 | 10 ±5.4 | Marketscan Database; 31. |
| Value 1 day lost ($)[g] | Indirect | 65 | 100 or 65 | 65 | 30 |
| Subtotal ($)[c] | | 3,366 | 6,842 | 7,653 | |
| **Outpatient visits** | | | | | |
| Avg. no. visits[h] | Direct | 1.52 | 1.52 | 1.52 | Marketscan Database |
| Most likely ± min or max net payment per visit($)[i] | Direct | 49 ±13 | 38 ± 12 | 50 ± 16 | Marketscan Database |
| Avg. copayment for outpatient visit ($) | Direct | 5 | 4 | 4 | Marketscan Database |
| Most likely ± min or max net payment per prescription($)[j] | Direct | 25 ± 18 | 36 ± 27 | 36 ± 22 | Marketscan Database |
| Avg. prescriptions per visit | Direct | 0.9 | 1.8 | 1.4 | Marketscan Database |
| Avg. copayment per prescription ($) | Direct | 3 | 3 | 3 | Marketscan Database |
| Days lost | Indirect | 3 | 2 | 5 | 4,5 |
| Value 1 day lost ($)[g] | Indirect | 65 | 100 | 65 | 30 |
| Subtotal ($)[c] | | 300 | 330 | 458 | |
| **Ill, no medical care sought** | | | | | |
| Days lost | Indirect | 3 | 2 | 5 | 4,5 |
| Value 1 day lost ($)[g] | Indirect | 65 | 100 | 65 | 30 |
| Over-the-counter drugs ($) | Direct | 2 | 2 | 2 | Assumed |
| Subtotal ($)[c] | | 197 | 202 | 327 | |

[a]Average present value (PV), using a 3% discount rate, of expected future lifetime earnings and housekeeping services, weighted by age and gender (30) and adjusted to 1995 dollars (by multiplying by a factor of 1.07) (16).
[b]Most likely, with ± defining the minimum and maximum costs for a triangular distribution (18) for Monte Carlo analysis (13-15). The values were calculated by using cost data from Marketscan Database (The MEDSTAT Group, Ann Arbor, MI) and multiplying it by a hospital cost-to-charge ratio of 0.53. The latter ratio is a weighted average of the urban and rural (urban = 0.80, rural = 0.20) cost-to-charge ratios calculated by the Health Care Finance Administration for August 1996 (31).
[c]Subtotals are the totals for each category of outcome, using the most likely estimates.
[d]Most likely, with minimum and maximum values of net payments for outpatient visits up to 14 days before admission date and up to 30 days after discharge date.
[e]Net payment for drug claims associated with outpatient visits up to 14 days before admission and up to 30 days after discharge.
[f]Most likely, with ± defining the minimum and maximum days lost due to hospitalization for a triangular distribution (18) for Monte Carlo analysis (13-15). Calculated using length of stay in hospital data from Marketscan Database (The MEDSTAT Group, Ann Arbor, MI) and adding a total of one additional day for convalescence and pre- and posthospitalization outpatient visits for 0-19 and 20-64 years of age. For 65 + years, two additional days were added to length of stay in hospital for convalescence and pre- and posthospitalization outpatient visits.
[g]For 0-19 and 65+ years age groups, a day lost to influenza was valued as equivalent to an unspecified day (30), denoting a value for time lost by care givers and family members related to taking care of a patient in these age groups. For 20-64 years of age, 60% of days lost due to hospitalizations and related convalescence and pre- and posthospitalization outpatient visits were valued as day off work ($100/day). The remaining 40% of days lost were valued as unspecified days ($65/day). For 20-64 years of age, when patients were not hospitalized at any point during their illness (i.e., outpatient status), all days lost were assumed days off work ($100/day).
[h]The number of visits per episode of influenza is an average across all age groups. From the database, it was found that 85% of all patients had less than three outpatient visits, with an average of 1.52 visits (Appendix II).
[i]Most likely, with minimum and maximum values of net payments for outpatient visits without any specified association to hospitalizations.
[j]Most likely, with ± defining the minimum and maximum cost per prescription, with the number of prescriptions per visit.

risk for influenza-related illness or death). Hospital costs attributed to pneumonia and bronchitis, acute bronchitis, chronic respiratory disease, and the identified heart conditions were then estimated as weighted averages (Appendix II).

The principal indirect cost was lost productivity, which was valued by using an age- and gender-weighted average wage (Table 3) (30). The economic cost of a death was valued at the present net value of the average expected future lifetime earnings, weighted for gender and age (30). All costs were standardized to 1995 US$ values.

The cost of fully vaccinating a person (i.e., administering the number of doses necessary to protect against disease) was modeled with two assumed values, approximately $21 and $62 per person fully vaccinated (Table 4). These costs include the cost of the vaccine, as well as its distribution and administration (health-care worker time, supplies); patient travel; time lost from work and other activities; and cost of side effects (including Guillain-Barré syndrome) (Table 4) (Appendix II).

### Vaccine Effectiveness

The assumed levels of vaccine effectiveness used to estimate the savings gained due to a vaccine-based intervention are described in Appendix I; the equation defining savings from outcomes averted contains the rate of compliance multiplied by the assumed vaccine effectiveness. In cases requiring two doses of vaccine to satisfactorily protect against influenza-related illness and death, a person was considered compliant only after both doses.

### Net Returns of Vaccination: Sensitivity Analyses

To illustrate the importance of the death rate in determining economic outcomes, we conducted further sensitivity analyses in which the death rates for persons not at high risk were one quarter or half of those used in the main analyses (Table 2).

### Insurance Premiums

To determine how much should be spent each year to plan, prepare, and practice to ensure that mass vaccinations can take place if needed, we considered the funding of those activities as an annual insurance premium (32). The premium would be used to pay for improving surveillance systems, ensuring sufficient supply of vaccine for high-priority groups (and possibly the entire U.S. population), conducting research to improve detection of new influenza subtypes, and developing emergency preparedness plans to ensure adequate medical care and maintenance of essential community services (32). We calculated the premium as follows (33): annual insurance premium = net returns from an intervention x the annual probability of a pandemic.

Table 4. Cost of vaccination[a] during an influenza pandemic, with specific costs assigned to side effects of vaccination

| Item | Probability of effect[b] | Cost of case of side effect ($)[b] | Lower-cost scenario ($/ patient) | Upper-cost scenario ($/ patient) |
|---|---|---|---|---|
| Assumed cost of vaccination[a] (excluding side effects) | | | 18 | 59 |
| Side effects | | | | |
| Mild[c] | 0.0325 | 94 | 3.05 | 3.05 |
| GBS[d] | 0.000002 | 100,800 | 0.20 | 0.20 |
| Anaphylaxis | 0.000000157 | 2,490 | 0.01 | 0.01 |
| Total cost per patient | | | 21.26 | 62.26 |

[a]The cost of vaccination includes the cost of the vaccine, the cost of administering the vaccine, value of time spent by a person traveling to and from the place of vaccination, and patient-associated travel costs. Included in the costs of the vaccine are any costs associated with the rapid production of a larger-than-usual number of doses and the rapid delivery and correct storage of doses at vaccination sites around the country. For $18, the costs were assumed to be $10 for vaccine + administration, $4 patient time (half hour), $4 patient travel costs. For $59, the costs were assumed to be $20 for vaccine + administration (this could include the cost of two doses), $32 patient time (two trips at 2 hours per trip), and $7 patient travel costs. For comparison, a review of 10 published articles found a range of $5 to $22 per dose of vaccine, with a medium [sic] cost of $14 per dose (10). Additional details are provided in the background paper (see Appendix II). These breakdowns are illustrations only of what might be deemed reasonable estimates of time and cost. Actual costs might vary substantially and will depend on the number of doses needed to achieve a satisfactory protective response, as well as the efficiency of giving vaccinations to millions of persons.

[b]Probabilities and average cost of treating each category of side effect were derived from (3).

[c]Mild side effects include sore arms due to vaccination, headaches, and other minor side effects that may require a visit to a physician or may cause the patient to miss 1 to 2 days of work.

[d]GBS = Guillain Barré syndrome.

### Vaccination Priorities and Distribution

During the early stages of a pandemic, the supply of influenza vaccine will likely be limited. Even if sufficient vaccine is produced to vaccinate the entire U.S. population, it will take time to administer the vaccine to all, especially if two doses are required. Because a pandemic will be caused by a new subtype of influenza, two doses of vaccine may be required. Who should receive priority for vaccination until vaccine supplies are more plentiful? To illustrate the use of the model in estimating the impact of different priorities, we created sample priority lists by using three different criteria: total deaths, risk for death, and maximizing net returns due to vaccination. In choosing the criteria for priorities, society must debate the main goal of a pandemic vaccination plan: prevent deaths, regardless of age and position in society; prevent deaths among those at greatest risk (i.e., 65 years of age); or minimize the social disruption. If the last is the goal of society, the net return due to vaccination should be used to set priorities.

The model can also be used to compare the economic consequences of plans that specify which target populations are vaccinated. To illustrate this capability, we constructed four options for prioritizing vaccine distribution. For Option A, the target population is similar to current Advisory Committee on Immunization Practices (ACIP) recommendations, with production and use of vaccine similar to current, intrapandemic recommendations (17). We assumed 77.4 million vaccinees.[4] Option B targets the number of vaccinees as outlined in Option A plus approximately 20 million essential service providers (5 million health-care workers and 15 million providers of other service) (99.2 million vaccinees). Option C aims to achieve a 40% effective coverage of the entire U.S. population (106.1 million vaccinees), and Option D, 60% effective coverage of the entire U.S. population (159.2 million vaccinees).

The number of vaccine doses required to meet each option will depend on the number of doses per person needed to obtain an immune response. If two are needed, lack of compliance with a two-dose regimen will mean that the actual number of doses needed will be higher than double the target population for each option (i.e., >40% or >60% of the population will have to receive the first dose to ensure that 40% or 60% are fully vaccinated). If two doses are required, the cost per person vaccinated will increase (Table 4).

### Findings

### Illnesses and Deaths

The number of hospitalizations due to an influenza epidemic ranged from approximately 314,000 (5th percentile = 210,000; 95th percentile = 417,000) at a gross attack rate of 15% to approximately 734,000 (5th percentile = 441,000; 95th percentile = 973,000) at a gross attack rate of 35% (Figure 1). The mean numbers of persons requiring outpatient-based care ranged from approximately 18 million (gross attack rate of 15%) to 42 million (gross attack rate of 35%) (Figure 1). The mean numbers of those clinically ill not seeking medical care but still sustaining economic loss ranged from approximately 20 million (gross attack rate of 15%) to 47 million (gross attack rate of 35%) (Figure 1). The estimated number of deaths ranged from approximately 89,000 (5th percentile = 55,000; 95th percentile = 122,000) at a gross attack rate of 15%, which increased to approximately 207,000 deaths (5th percentile = 127,000; 95th percentile = 285,000) at a gross attack rate of 35% (Figure 1).

Groups at high risk (approximately 15% of the total U.S. population) (Table 1) would likely be disproportionately affected by an influenza pandemic. These groups accounted for approximately 85% of all deaths, with groups at high risk in the 20- to 64-year-old age group accounting for approximately 41% of total deaths (Table 5). Groups at high risk also accounted for 38% of all hospitalizations and 20% of all outpatient visits (Table 5).

### Economic Impact of an Influenza Pandemic

Without large-scale immunization, the estimates of the total economic impact in the United States of an influenza pandemic ranged from $71.3 billion (5th percentile = $35.4 billion; 95th percentile = $107.0 billion) (gross attack rate of 15%) to $166.5 billion (5th percentile = $82.6 billion; 95th percentile = $249.6 billion) (gross attack rate of 35%) (Table 6). At any given attack rate, loss of life accounted for approximately 83% of all economic losses. Outpatients, persons ill but not seeking medical care, and inpatients accounted for approximately 8%, 6%, and 3%, respectively, of all economic losses (Table 6) (Appendix II).

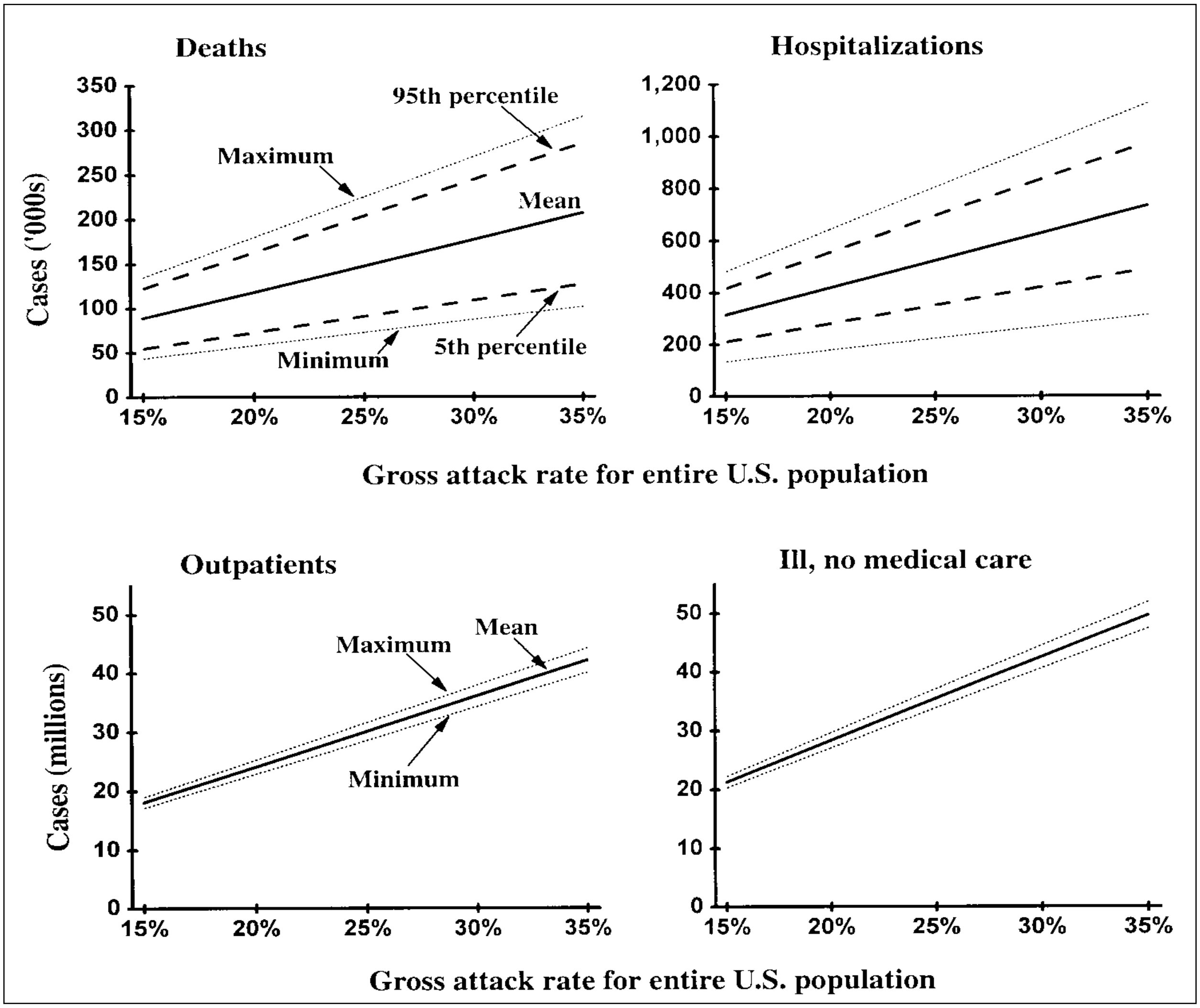

Figure 1: Impact of influenza pandemic in the United States: mean, minimum, maximum, and 5th and 95th percentiles of total death, hospitalizations, outpatients, and those ill (but not seeking medical care) for different gross attack rates. Note that for each gross attack rate, data are totals for all age groups and risk categories.

Table 5. Impact, by age group, death, hospitalizations, and outpatients accounted for by groups at high risk during an influenza pandemic[a]

| Category | Age group (yrs) | Total cases at high risk (%) Mean | 5th | 95th |
|---|---|---|---|---|
| Death | 0-19 | 9.0 | 1.4 | 20.2 |
| | 20-64 | 40.9 | 11.1 | 60.9 |
| | 65 + | 34.4 | 22.7 | 52.1 |
| | Total | 84.3 | | |
| Hospitalizations | 0-19 | 4.6 | 2.1 | 7.9 |
| | 20-64 | 14.7 | 7.4 | 23.4 |
| | 65 + | 18.3 | 11.0 | 27.6 |
| | Total | 37.6 | | |
| Outpatients | 0-19 | 5.0 | 4.7 | 5.4 |
| | 20-64 | 10.4 | 9.8 | 11.0 |
| | 65 + | 4.0 | 3.9 | 4.2 |
| | Total | 19.5 | | |

[a]See Table 1 for distribution of groups at high and not at high risk within the U.S. population.

### Net Value of Vaccination

If it cost $21 to vaccinate a person and the effective coverage were 40%, net savings to society would result from vaccinating all age and risk groups (Figure 2). However, vaccinating certain age and risk groups rather than others would produce higher net returns. For example, vaccinating patients ages 20 to 64 years of age not at high risk would produce higher net returns than vaccinating patients ages 65 years of age and older who are at high risk (Figure 2). At a cost of $62 per vaccinee and gross attack rates of less than 25%, vaccinating populations at high risk would still generate positive returns (Figure 2). However, vaccinating populations not at high risk would result in a net loss (Figure 2).

Table 6. Costs (direct and indirect) of influenza pandemic per gross attack rate:[a] deaths, hospitalizations, outpatients, illnesses, and total costs (in 1995 US$)

| | Cost per gross attack rate ($ millions) | | | | |
|---|---|---|---|---|---|
| | 15% | 20% | 25% | 30% | 35% |
| Deaths | | | | | |
| Mean | 59,288 | 79,051 | 98,814 | 118,577 | 138,340 |
| 5th percentile | 23,800 | 31,733 | 39,666 | 47,599 | 55,532 |
| 95th percentile | 94,907 | 126,543 | 158,179 | 189,815 | 221,451 |
| Hospitalizations | | | | | |
| Mean | 1,928 | 2,571 | 3,214 | 3,856 | 4,499 |
| 5th percentile | 1,250 | 1,667 | 2,084 | 2,501 | 2,917 |
| 95th percentile | 2,683 | 3,579 | 4,472 | 5,367 | 6,261 |
| Outpatients | | | | | |
| Mean | 5,708 | 7,611 | 9,513 | 11,416 | 13,318 |
| 5th percentile | 4,871 | 6,495 | 8,119 | 9,742 | 11,366 |
| 95th percentile | 6,557 | 8,742 | 10,928 | 13,113 | 15,299 |
| Ill, no medical care sought[b] | | | | | |
| Mean | 4,422 | 5,896 | 7,370 | 8,844 | 10,317 |
| 5th percentile | 3,270 | 4,360 | 5,450 | 6,540 | 7,629 |
| 95th percentile | 5,557 | 7,409 | 9,262 | 11,114 | 12,967 |
| Grand totals | | | | | |
| Mean | 71,346 | 95,128 | 118,910 | 142,692 | 166,474 |
| 5th percentile | 35,405 | 47,206 | 59,008 | 70,810 | 82,611 |
| 95th percentile | 106,988 | 142,650 | 178,313 | 213,975 | 249,638 |

[a]Gross attack rate = percentage of clinical influenza illness per population.
[b]Persons who become clinically ill due to influenza but do not seek medical care; illness has an economic impact (e.g., half day off work).

### Sensitivity Analyses

At a vaccination cost of $21.26 per vaccinee, reducing the death rates to half and one quarter of the initial values (Table 2) left positive mean net returns for all age groups not at high risk. However, at a vaccination cost of $62.26 per vaccinee, reducing death rates to half and one quarter of the initial values resulted in negative mean net returns for all age groups not at high risk. The results are much less sensitive to increases in gross attack rate than to increases in death rate. For example, assuming a cost of $62.26 per vaccinee and death rates that are half the initial rates, increasing the gross attack rate from 15% to 25% still resulted in negative net returns for all age groups, regardless of assumed level of vaccine effectiveness.

### Implications for Policy

The amount of the insurance premium to spend on planning, preparedness, and practice for responding to the next influenza pandemic ranged from $48 million to $2,184 million per year (Table 7). The amount was sensitive to the probability of the pandemic, the cost of vaccinating a person, and the gross attack rate. Because higher costs of vaccination reduce net returns from an intervention, increased vaccination costs reduced the premiums. Conversely, increases in gross attack rates (all other inputs held constant) increased the potential returns from an intervention and thus the amount of premiums.

When risk for death is used as the criterion for who will be vaccinated first, persons ages 65 years and older receive top priority (Table 8); however, when mean net returns due to vaccination are used as the criterion, that group receives the lowest priority (Table 8). Regardless of criteria used, persons at high risk ages 0 to 19 and 20 to 64 years would always receive priority over persons not at high risk from the same age groups (Table 8).

While Option A would ensure positive mean net returns, Option B would result in greater mean net returns (Figure 3). Changing the strategy from vaccinating specific groups (Option B) to vaccinating 40% of the population decreased mean net returns (Figure 3). Only Option D resulted in higher mean net returns

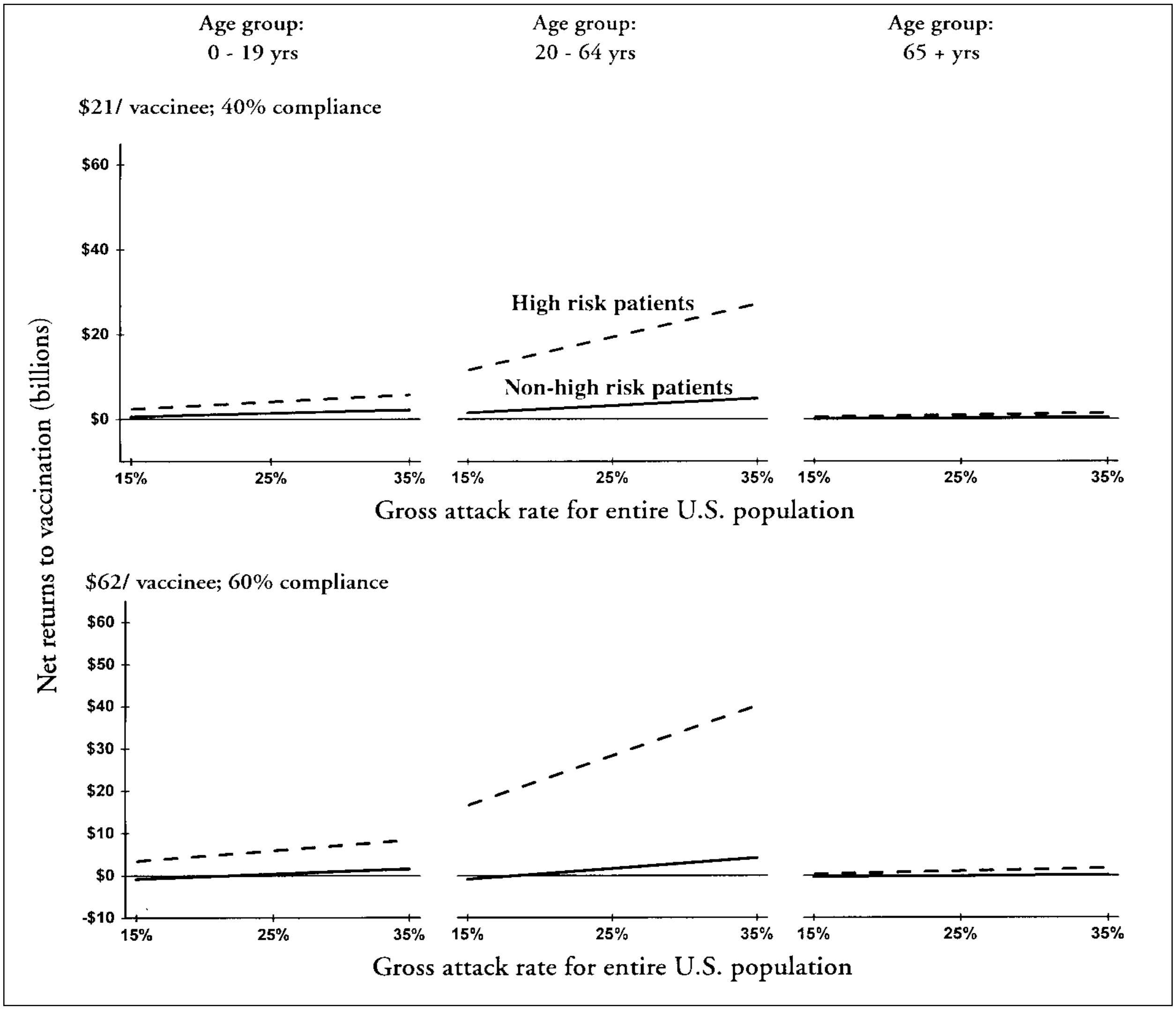

Figure 2: Mean net returns due to vaccination, by age group, for different gross attack rates and percentages of compliance. Case-age distributions are given in Table 1. Assumed vaccine effectiveness is the same as the high vaccine effectiveness defined in Appendix I.

Table 7. The mean annual insurance premium[a] for planning, preparing, and practicing to respond to the next influenza pandemic

| | | Mean (s.d.) insurance premium ($ millions) | | | | | |
|---|---|---|---|---|---|---|---|
| | | Low vaccine effectiveness[b] x 40% compliance | | | High vaccine effectiveness[b] x 60% compliance | | |
| | | Probability of pandemic | | | Probability of pandemic | | |
| Gross attack rate | Cost of vaccination per vaccinee($) | 1 in 30 years | 1 in 60 years | 1 in 100 years | 1 in 30 years | 1 in 60 years | 1 in 100 years |
| 15% | 21 | 306 (122) | 153 (61) | 92 (37) | 872 (341) | 435 (170) | 262 (103) |
| | 62 | 162 (122) | 81 (61) | 48 (37) | 654 (341) | 326 (170) | 196 (103) |
| 25% | 21 | 561 (204) | 280 (102) | 168 (61) | 1,528 (569) | 762 (284) | 459 (171) |
| | 62 | 416 (204) | 207 (102) | 125 (61) | 1,311 (569) | 653 (284) | 394 (171) |
| 35% | 21 | 815 (286) | 406 (142) | 245 (86) | 2,184 (796) | 1,089 (397) | 656 (239) |
| | 62 | 670 (286) | 334 (142) | 201 (86) | 1,967 (796) | 980 (397) | 591 (239) |

[a]Defined here as the amount of money to be spent each year to plan, prepare, and practice to ensure that such mass vaccinations can take place if needed. See text for description of calculating premiums. The mathematically optimal allocation of such funds for each activity requires a separate set of calculations.
[b]Low and high levels of vaccine effectiveness are defined in Appendix I.

than Option B. Note, however, that the 5th and 95th percentiles for each option overlapped with those of other options. Thus, the differences in mean values between the options may not occur in practice.

### Conclusions

### Impact of an Influenza Pandemic

Although the next influenza pandemic in the United States may cause considerable illness

Table 8. Setting vaccination priorities: Which age group or group at risk should be vaccinated first?

| | Criteria for prioritization | | |
|---|---|---|---|
| Priority | Risk for death[a] | Total deaths[b] | Returns due to vaccination |
| 1 (top) | High risk 65 + yrs | High risk 20 - 64 yrs | High risk 20 - 64 yrs |
| 2 | Not at high risk 65 + yrs | High risk 65 + yrs | High risk 0 - 19 yrs |
| 3 | High risk 0 - 19 yrs | High risk 0 - 19 yrs | Not at high risk 20 - 64 yrs |
| 4 | High risk 20 - 64 yrs | Not at high risk, 65 + yrs | Not at high risk 0 - 19 yrs |
| 5 | Not at high risk 20 - 64 yrs | Not at high risk 20 - 64 yrs | High risk 65 + yrs |
| 6 (bottom) | Not at high risk 0 - 19 yrs | Not at high risk 0 - 19 yrs | Not at high risk 65 + yrs |

[a]Priorities set by risk for death are set according to lower-limit estimates of deaths per 1,000 population for each age and risk group.
[b]The priority list using the total deaths criteria was set by examining the percentage of total deaths that each age and risk group contributed to the total deaths estimated due to a pandemic. The group with the highest percentage (i.e, contributes the largest number of deaths) is listed as having the highest priority.

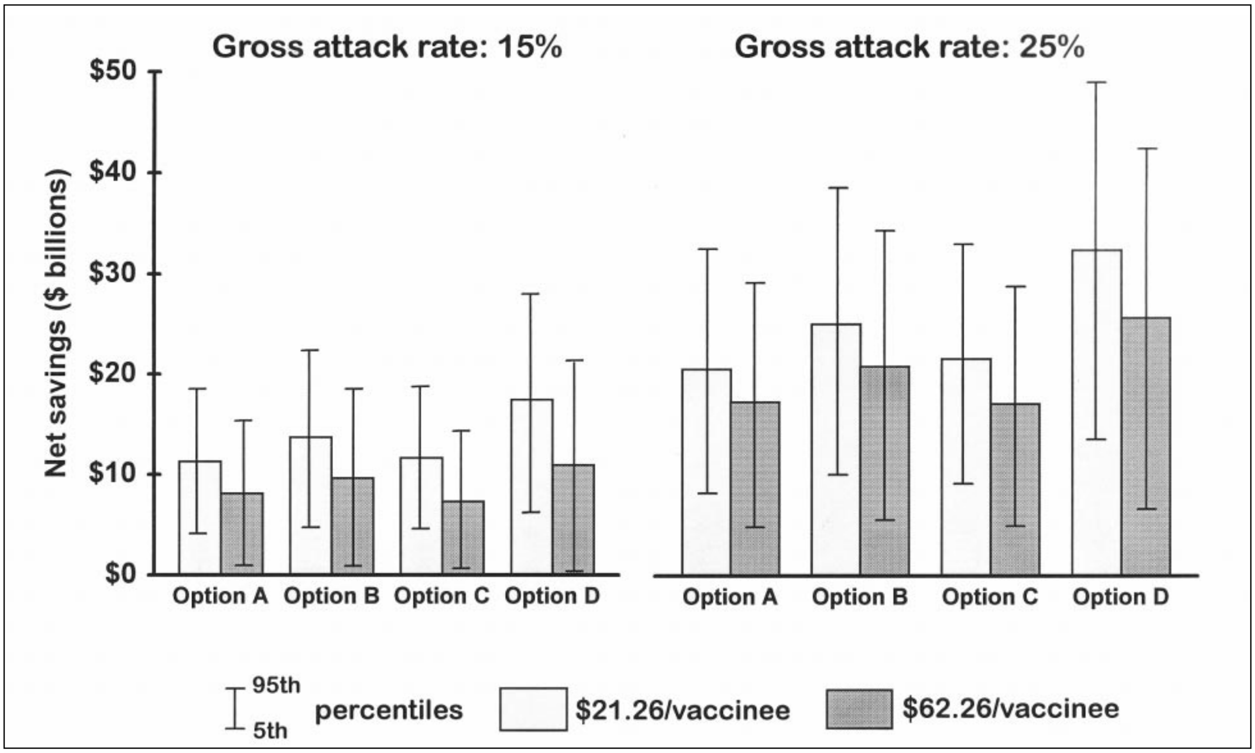

Figure 3: Four options for responding to an influenza pandemic: mean net economic returns. Notes: a) Bars show mean net returns for each option and assumed cost of vaccination. b) Option A: Similar to current Advisory Committee on Immunization Practices recommendations, with production and use similar to current, intra-pandemic recommendations (17). Assumed approximately 77 million vaccinees. Option B: Number of vaccinees as outlined in Scenario A plus 20 million essential service providers (5 million health-care workers + 15 million other service providers). Option C: Aim to achieve a 40% coverage of total U.S. population. Option D: Aim to achieve 60% coverage of total U.S. population (Appendix II).

and death (Figure 1), great uncertainty is associated with any estimate of the pandemic's potential impact. While the results can describe potential impact at gross attack rates from 15% to 35%, no existing data can predict the probability of any of those attack rates actually occurring. In addition, the groups at high risk are likely to incur a disproportionate number of deaths (Table 5); 50% or more of the deaths will likely occur among persons age 65 years and older (Appendix II), a distribution also found in the influenza pandemics of 1918, 1957, and 1968 (2).

Our results illustrate that the greatest economic cost is due to death (Table 6). Therefore, all other things being equal, the largest economic returns will come from the intervention(s) that prevents the largest number of deaths. A limitation of the model is that, beyond the value of a lost day of work (Table 3), the model does not include any valuation for disruptions in commerce and society. For example, if many long-distance truck drivers were unavailable to drive for 1 or 2 weeks, there might be disruptions in the distribution of perishable items, especially food. These multiplier effects are not accounted for in this model, mainly because an estimate of an appropriate multiplier will depend on who becomes ill, how many become ill, when they become ill, and for how long they are ill.

All other factors being held constant, the net returns due to vaccination are sensitive to the combination of price and gross attack rate, with some scenarios generating negative mean returns (Figure 2). Further, some scenarios with a positive mean net return had a negative 5th percentile (Appendix II). The fact that negative results can be generated should serve as a warning that many interventions may not guarantee a net positive economic return.

### Implications for Policy

The premium that could be spent each year for influenza pandemic response (planning, preparedness, and practice) depends most on the assumed probability of the pandemic (Table 7). The wide range in premiums presents a cautionary tale of the difference between possibility and probability of an influenza pandemic. What cannot be stated with any certainty are the probability of a pandemic and the number of persons who will become ill and die. Deciding the difference between possibility and probability was a key decision point in the swine flu incident of 1976-77 (34).

Vaccination priorities depend on the objectives. If preventing the greatest number of deaths is the most important goal, society should ensure that those in the groups at high risk become vaccinated first, followed by those age 65 years or older who have no preexisting medical conditions making them more susceptible to complications from influenza (Table 8). However, if maximizing economic returns is the highest priority, persons 0 to 64 years of age, regardless of risk, should be vaccinated first (Table 8). Results also illustrate the need to be precise in defining the criterion used for setting priorities. For example, stating that preventing death will be the criteria used is not sufficiently precise because different priority lists can be drawn up using death rates versus total deaths (Table 8).

The criteria used to generate the results in Table 8 do not define the entire set of possible methods of setting priorities. Society may decide to use another criterion or set of criteria. Priorities for vaccination may also depend on the epidemiology of the pandemic. For example, if the strain causing the pandemic were particularly virulent among those ages 20 to 40 years, that age group may receive highest priority. Since the epidemiology of the next pandemic is unknown, any plan must allow flexibility in determining criteria for setting priorities. Table 8 provides a starting point for debate regarding who should be vaccinated first.

The net returns for the four scenarios modeled (Figure 3) further illustrate the need to clearly set criteria, goals, and objectives for a vaccine-based intervention for the next influenza pandemic. Some may state that Options C and D represent a more egalitarian means of distributing vaccine. However, egalitarianism would cost society more since the mean net returns from Options C are lower than those from Option B (Figure 3). Option D produces higher returns than Option B (Figure 3), but vaccinating 60% of the U.S. population in a short time would be difficult, especially if two doses of vaccine are required. If two doses were required, Option D would mean producing, delivering, and administering approximately 320 million doses of vaccine in a 2- to 3-month period, which has never been accomplished in the United States.

### Acknowledgments

We thank Nancy Arden, Rob Breiman, Bill Jordan, Marty Meyers, Alicia Postema, Steve Schoenbaum, Larry Schonberger, Larry Sparks, and Ray Strikas for their help and encouragement.

Dr. Meltzer is senior health economist, Office of the Director, National Center for Infectious Diseases, Centers for Disease Control and Prevention. His research interests focus on assessing the economics of public health interventions such as oral raccoon rabies vaccine, Lyme disease vaccine, and hepatitis A vaccine, as well as estimating the economic burden of bioterrorism, dengue, pandemic influenza, and other infectious diseases. His research uses various methods, including Monte Carlo modeling, willingness-to-pay surveys (contingent valuation), and the use of nonmonetary units of valuation, such as Disability Adjusted Life Years.

### References

1. Patriarca PA, Cox NJ. Influenza pandemic preparedness plan for the United States. J Infect Dis 1997;176 Suppl 1:S4-7.
2. Simonsen L, Clarke MJ, Schonberger LB, Arden NH, Cox NJ, Fukuda K. Pandemic versus epidemic influenza mortality: a pattern of changing age distribution. J Infect Dis 1998;178:53-60.
3. Office of Technology Assessment, U.S. Congress. Cost effectiveness of influenza vaccination. Washington: Government Printing Office; 1981.
4. Kavet J. A perspective on the significance of pandemic influenza. Am J Public Health 1977;67:1063-70.
5. Campbell DS, Rumley MA. Cost-effectiveness of the influenza vaccine in a healthy, working-age population. J Occup Environ Med 1997;39:408-14.

### Appendix I

For the equation in the main text defining net returns due to vaccinations, savings from outcomes averted and the costs of vaccination are calculated as follows:

$$\text{Savings from outcomes averted}_{\text{age, risk group}} = \sum_{\text{Outcomes}} (\text{Number with outcome}_{\text{death, hospitalization, outpatient, ill, no medical care}} \text{ before intervention}_{\text{age, risk group}}$$

$$\times \text{ compliance}_{\text{age, risk group}} \times \text{ vaccine effectiveness}_{\text{Outcomes}} \times \text{ \$value of outcome}_{\text{death, hospitalization, outpatient, ill, no medical care}} \text{ prevented})$$

and;

$$\text{Cost of vaccination}_{\text{age, risk group}} = \text{\$cost/vaccinee x population}_{\text{age risk group}} \times \text{ compliance}_{\text{age, risk group}}$$

Table: High and low levels of assumed vaccine effectiveness

| | Vaccine effectiveness in preventing disease outcomes[ab] | | | | | |
|---|---|---|---|---|---|---|
| | High[c] | | | Low[c] | | |
| Disease outcomes | 0-19 yrs | 20-64 yrs | 65+ yrs | 0-19 yrs | 20-64 yrs | 65+ yrs |
| Death | 0.70 | 0.70 | 0.60 | 0.40 | 0.40 | 0.30 |
| Hospitalization | 0.55 | 0.55 | 0.50 | 0.55 | 0.55 | 0.50 |
| Outpatient visits | 0.40 | 0.40 | 0.40 | 0.40 | 0.40 | 0.40 |
| Ill, no medical care sought | 0.40 | 0.40 | 0.40 | 0.40 | 0.40 | 0.40 |

[a]Vaccine effectiveness is defined as the reduction in the number of cases in each of the age and disease categories.
[b]Within a defined age group, it was assumed that there was no difference in vaccine effectiveness between subgroups at high risk and not at high risk.
[c]The terms high and low level of effectiveness are subjective and reflect only a judgment of the levels of effectiveness in the two scenarios relative to each other.

### Appendix II

A background paper, containing additional methodological details and results, is available electronically at the following URL: http.www.cdc.gov/ncidod/EID/vol5no5/meltzerback.htm.

6. Carrat F, Valleron A-J. Influenza mortality among the elderly in France, 1980-90: how many deaths may have been avoided through vaccination? J Epidemiol Community Health 1995;49:419-25.
7. Riddiough MA, Sisk JE, Bell JC. Influenza vaccination: cost-effectiveness and public policy. JAMA 1983;249:3189-95.
8. Patriarca PA, Arden NH, Koplan JP, Goodman RA. Prevention and control of type A influenza infections in nursing homes: benefits and costs of four approaches using vaccination and amantadine. Ann Intern Med 1987;107:732-40.
9. Schoenbaum SC. Economic impact of influenza: the individuals perspective. Am J Med 1987;82 Supp 6A:26-30.
10. Jefferson T, Demicheli V. Socioeconomics of influenza. In: Nicholson KG, Webster RG, Hay AJ, editors. Textbook of influenza. London (UK): Blackwell Science; 1998. p. 541-7.
11. Schoenbaum SC, McNeil BJ, Kavet J. The swine-influenza decision. N Eng J Med 1976;295:759-65.
12. Cliff AD, Haggett P. Statistical modelling of measles and influenza outbreaks. Stat Methods Med Res 1993;2:43-73.
13. Critchfield GC, Willard KE. Probabilistic analysis of decision trees using Monte Carlo simulation. Med Decis Making 1986;6:85-92.
14. Dobilet P, Begg CB, Weinstein MC, Braun P, McNeil BJ. Probabilistic sensitivity analysis using Monte Carlo simulation: a practical approach. Med Decis Making 1985;5:157-77.
15. Dittus RS, Roberts SD, Wilson JR. Quantifying uncertainty in medical decisions. J Am Coll Cardiol 1989;14:23A-8.
16. U.S. Bureau of the Census. Statistical abstract of the United States: 1997. 117th ed. Washington: The Bureau; 1997.
17. Centers for Disease Control and Prevention. Prevention and control of influenza: recommendations of the Advisory Committee on Immunization Practices (ACIP). MMWR Morb Mortal Wkly Rep 1998;47(RR-6):1-26.
18. Evans M, Hastings N, Peacock B. Statistical distributions. 2nd ed. New York: John Wiley; 1993.
19. Glezen PW. Emerging infections: pandemic influenza. Epidemiol Rev 1996;18:64-76.
20. Mullooly JP, Barker WH. Impact of type A influenza on children: a retrospective study. Am J Public Health 1982;72:1008-16.
21. Barker WH, Mullooly JP. Impact of epidemic type A influenza in a defined adult population. Am J Epidemiol 1980;112:798-813.
22. Simonsen L, Clarke MJ, Williamson GD, Stroup DF, Arden NH, Schonberger LB. The impact of influenza epidemics on mortality: introducing a severity index. Am J Public Health 1997;87:1944-50.
23. Fox JP, Hall CE, Cooney MK, Foy HM. Influenzavirus infections in Seattle families, 1975-1979. I. Study design, methods and the occurrence of infections by time and age. Am J Epidemiol 1982;116:212-27.
24. Glezen WP, Decker M, Joseph SW, Mercready RG. Acute respiratory disease associated with influenza epidemics in Houston, 1981-1983. J Infect Dis 1987;155:1119-26.
25. Serfling RE, Sherman Il, Houseworth WJ. Excess pneumonia-influenza mortality by age and sex in three major influenza A2 epidemics, United States, 1957-58, 1960 and 1963. Am J Epidemiol 1967;86:433-41.
26. Barker WH, Mullooly JP. Pneumonia and influenza deaths during epidemics: implications for prevention. Arch Intern Med 1982;142:85-9.
27. Glezen WP, Payne AA, Snyder DN, Downs TD. Mortality and influenza. J Infect Dis 1982;146:313-21.
28. McBean AM, Babish JD, Warren JL. The impact and cost of influenza in the elderly. Arch Intern Med 1993;153:2105-11.
29. Barker WH. Excess pneumonia and influenza associated hospitalization during influenza epidemics in the United States, 1970-78. Am J Public Health 1986;76:761-5.
30. Haddix AC, Teutsch SM, Shaffer PA, Dunet DO. Prevention effectiveness. New York: Oxford University Press; 1996.
31. The Federal Register. Vol 61, No. 170; 1996 Aug 30; p. 46301-2.
32. Kaufmann AF, Meltzer MI, Schmid GP. The economic impact of a bioterrorist attack: are prevention and postattack intervention programs justifiable? Emerg Infect Dis 1997;3:83-94.
33. Robinson LJ, Barry PJ. The competitive firm's response to risk. New York: Macmillian; 1987.
34. Neustadt RE, Fineberg HV. The swine flu affair: decision making on a slippery disease. Washington: U.S. Department of Health Education, and Welfare; 1978.
